# Establishment of a Rhesus Macaque Model for Scrub Typhus Transmission: Pilot Study to Evaluate the Minimal *Orientia tsutsugamushi* Transmission Time by *Leptotrombidium* *chiangraiensis* Chiggers

**DOI:** 10.3390/pathogens10081028

**Published:** 2021-08-13

**Authors:** Piyada Linsuwanon, Sirima Wongwairot, Nutthanun Auysawasdi, Taweesak Monkanna, Allen L. Richards, Surachai Leepitakrat, Piyanate Sunyakumthorn, Rawiwan Im-Erbsin, Katie Poole-Smith, Patrick McCardle

**Affiliations:** 1 Department of Entomology, USAMD-AFRIMS, Bangkok 10330, Thailand; sirimaw.ca@afrims.org (S.W.); nutthanuna@afrims.org (N.A.); taweesakm.fsn@afrims.org (T.M.); surachail.fsn@afrims.org (S.L.); betty.poolesmith.mil@afrims.org (K.P.-S.); patrick.mccardle.mil@afrims.org (P.M.); 2 Department of Preventive Medicine and Biostatistics, Uniformed Services University of Health Sciences, Bethesda, MD 20814, USA; allen.richards@comcast.net; 3 Department of Veterinary Medicine, USAMD-AFRIMS, Bangkok 10330, Thailand; piyanates.fsn@afrims.org (P.S.); rawiwani.fsn@afrims.org (R.I.-E.)

**Keywords:** scrub typhus, chigger transmission, scrub typhus disease model, transmission time

## Abstract

Recently, an intradermal inoculation of the rhesus macaque model of scrub typhus has been characterized at our institution. The current project was to establish a rhesus macaque model of scrub typhus using the naturally infected chigger challenge method that faithfully mimics the natural route of pathogen transmission to fully understand the host-pathogen-vector interactions influencing pathogen transmission. Unlike the needle-based inoculation route, *Orientia tsutsugamushi*-infected chiggers introduce both pathogen and chigger saliva into the host epidermis at the bite site. However, information on the interaction or influence of chigger saliva on pathogenesis and immunity of host has been limited, consequently hindering vaccine development and transmission-blocking studies. To characterize chigger inoculated *O. tsutsugamushi* in rhesus macaques, we determined the minimum chigger attachment time required to efficiently transmit *O. tsutsugamushi* to the immunocompetent hosts and preliminary assessed clinical parameters, course of bacterial infection, and host’s immunological response to identifying potential factors influencing pathogen infection. Chigger infestation on hosts resulted in: (i) Rapid transmission of *O. tsutsugamushi* within 1 h and (ii) antigen-specific type I and II T-cell responses were markedly increased during the acute phase of infection, suggesting that both systems play critical roles in response to the pathogen control during the primary infection. In summary, we demonstrate that *O. tsutsugamushi* infection in rhesus macaques via chigger challenge recapitulates the time of disease onset and bacteremia observed in scrub typhus patients. Levels of proinflammatory cytokines and chemokines were positively correlated with bacteremia.

## 1. Introduction

Scrub typhus is a neglected acute febrile illness caused by members of the genus *Orientia.* These pathogens are Gram-negative rod-shaped obligate intracytosolic bacteria belonging to the family *Rickettsiaceae* (*Proteobacteria* α-subdivision) [1,2]. *Orientia tsutsugamushi* (*O. tsutsugamushi*), endemic to Asia–Pacific region (Tsutsugamushi Triangle), *Candidatus* Orientia chuto found in the Middle East and Africa, and *Candidatus* Orientia chiloensis (described in over 50 scrub typhus cases in Chile) are responsible for scrub typhus around the world with the potential of new endemic areas and orientiae to be identified in the future [1,2,3,4]. The orientiae are introduced to warm-blooded animal hosts and humans through the bite of mite larvae (also known as chiggers) of the family *Trombiculidae.* Recent evidences suggest different chigger species play important roles in certain geographic regions, such as chiggers of the genus *Leptotrombidium* are efficient vectors of scrub typhus in the Asia–Pacific region [5,6,7,8], while *Microtrombicula* and *Herpatacarus* potentially play important roles in the pathogen transmission cycle in Africa and South America, respectively [3,9]. Comprehensive studies of genetic diversity and geographic distribution of the chigger are warranted to better understand the role of vectors and the risk of diseases they transmit.

Scrub typhus is characterized by nonspecific signs and symptoms, which can range from self-limited to life-threatening disease involving multi-organ failure and even fatality in some cases [10,11]. Patients commonly present with acute febrile illness, severe headache, body aches, and myalgia during the first 7 to 10 days after the bite from infected chiggers. Classically, a localized cutaneous necrotic lesion at the site of a chigger bite, called an eschar, may occur. However, the prevalence of eschar formation varies depending on geographic area and patient demography [12,13]. Other signs and symptoms of the infection include: rash, regional lymphadenopathy, hepatomegaly, splenomegaly, abdominal pain, and central nervous system involvement [14,15,16,17]. If diagnosed early and accurately, scrub typhus can be effectively treated with doxycycline, azithromycin, rifampicin, chloramphenicol, and derivatives [18]. Because signs and symptoms of the disease are often nonspecific, scrub typhus can be difficult to differentiate from other diseases that are cocirculated in the disease endemic areas, such as dengue fever, murine typhus, or leptospirosis, contributing to misdiagnosis and delayed treatment. Scrub typhus has a mortality rate of up to 70% in untreated patients and 2% in treated patients, with an average mortality of 6% [19]. Complications associated with scrub typhus are typically higher than dengue virus infection or malaria [20,21,22].

No broadly protective, long-lasting vaccine is currently available to prevent scrub typhus. The high immunogenic diversity of the pathogen limits long-lasting heterologous protection both naturally and by vaccine candidates. In addition, the lack of reliable disease models has critically hampered vaccine development and transmission-blocking studies [23,24]. Aside from murine models, several species of old-world monkeys have been the most widely accepted experimental disease model for scrub typhus since 1918. However, these nonhuman primate (NHP) models have not significantly evolved since their inception [25]. Clinical and immune responses to scrub typhus have been studied in three species of monkeys indigenous to endemic areas of the disease: (i) silvered leaf monkey (*Presbytis cristatus*), (ii) cynomolgus monkey (*Macaca fasicularis*), and (iii) rhesus macaque (*Macaca mulatta*). In silvered leaf monkeys, the incubation period of scrub typhus is 4 to 10 days, and disease onset is marked by fever and lethargy. Eschar at the inoculation site may develop, but other clinical hallmarks, including generalized lymphadenopathy, splenomegaly, and hepatomegaly, have not been reported. Infection by different strains of *O. tsutsugamushi* produced different courses of infection and clinical signs [26,27,28,29,30,31]. A major challenge of using the silvered leaf monkey as an experimental model is the difficulty in maintaining this species in captivity, including high nonspecific mortality rates that are frequently reported. To overcome these difficulties, subsequent research evaluated cynomolgus monkeys as a scrub typhus model of choice [32,33,34,35]. Following *O. tsutsugamushi* infection, cynomolgus monkeys developed clinical signs similar to those in silvered leaf monkeys, resembling human infection, but produced significantly higher specific antibody responses. However, the immunized cynomolgus monkeys failed to generate long-term protective immunity and developed rickettsemia after being rechallenged, whether with the homologous or heterologous strains of *O. tsutsugamushi* [29]. This observation indicates that the lack of immunologic memory would make this model unsuitable for long-lasting scrub typhus vaccine study.

Rhesus macaques are the most widely used nonhuman primates in biomedical research, and recent animal models of scrub typhus have been developed using this species. Sunyakunthorn and colleagues demonstrated scrub typhus disease progression in rhesus macaques and reported that the infected rhesus macaques developed more rapid signs of disease onset than cynomolgus monkeys [36]. All infected rhesus macaques developed uniformly severe eschar lesions at inoculation sites, followed by regional lymphadenopathy. Strong cell-mediated lymphocytes and macrophages and specific immunoglobulin (Ig) M and IgG responses to *O. tsutsugamushi* infection were observed in all infected rhesus macaques. Additionally, the pathology of the experimental rhesus macaques at the terminal stage of infection was similar to the characteristics observed in scrub typhus patients. Based on the availability, ease of maintenance, and demonstrable clinical and immunological responses of rhesus macaques following experimental infection with *O. tsutsugamushi*, this species may represent a promising candidate to study pathological and immunological responses to *O. tsutsugamushi* infection, as well as become a suitable model for evaluation of vaccine efficacy.

Recently, nearly all available animal models of scrub typhus have been developed based on needle inoculation of *O. tsutsugamushi*. This route of pathogen transmission is vastly different from the vector-mediated natural infection and may lead to erroneous results of challenge studies. In comparison with other vector-borne disease models, studies of mosquito-borne and tick-borne diseases suggested that the needle-based transmission route does not fully recapitulate vector-mediated transmission, due to the absence of important immunomodulatory molecules contained in vector saliva [37,38] or by bypassing the natural route of host cell invasion which alters the resulting host immune response. Utilization of needle-based inoculation approach potentially leads to incomparable results to the vector-mediated transmission and immunological response observed in humans [39,40,41,42].

In contrast to needle-based inoculation, chiggers feed intradermally and can transmit *O. tsutsugamushi* along with the salivary proteins to an anatomically precise target in the epidermis or dermis of the host skin through their hyaline tube-like feeding structures called stylostomes. Chiggers do not have needle-like mouthparts like other hematophagous arthropods. To feed, chiggers inject complex salivary secretions containing lytic enzymes and anti-coagulants homologs of tick cement proteins to break down host epidermal cells [43]. The process results in the compartmentalization of cellular contents and formation of the stylostome that extends into the host epidermis [44]. Stylostome serves as a funnel to acquire liquified tissue as well as a route of pathogen transmission. After being introduced into the host, *O. tsutsugamushi* systemically disseminate to local lymph nodes and then to distant organs via the lymphatic system. Similar to other vectors, chigger saliva is believed to contain various substances that modulate host immunity at the bite site to overcome host defense and facilitate pathogen infection. Recent experimental data from our group demonstrated that mice infected with *O. tsutsugamushi* through the chigger challenge developed earlier onset of symptoms than mice infected through needle-challenge (unpublished observations), suggesting that chigger-based transmission influenced the disease progression and chigger saliva potentially play an important role in facilitating pathogen dissemination. However, our previous study initially focused on the pathogenesis at the chronic stage, moribund condition [45]. The effects of chigger saliva on *O. tsutsugamushi* infection, pathogenesis, and kinetics of cell-mediated and humoral immune responses have not been rigorously studied in either murine or rhesus macaque models.

To overcome these knowledge gaps, our goal was to establish a well-validated chigger transmission-based scrub typhus disease model using rhesus macaques. In the initial phase of the study, we aimed to determine the minimum attachment time required for *Leptotrombidium chiangraiensis* (Tanskul and Linthicum) chiggers to successfully transmit *O. tsutsugamushi* to rhesus macaque hosts and to better understand the course of primary infection following chigger challenge. Knowledge gained from our pilot study provided preliminary criteria to determine the minimum restraining time for the experimental animal and optimum site for chigger challenge which allow us to efficiently assesse the host’s clinical and immune responses to primary infection. This model can be used to begin to examine some of the fundamental unsolved issues with regard to the host-pathogen-vector interactions and host immunological and pathological response to *O. tsutsugamushi* infection via chigger bite.

## 2. Results

### 2.1. Bacterial Transmission to Rhesus Macaques Occurs Rapidly within 1 h of Chigger Feeding

**Figure 1:**
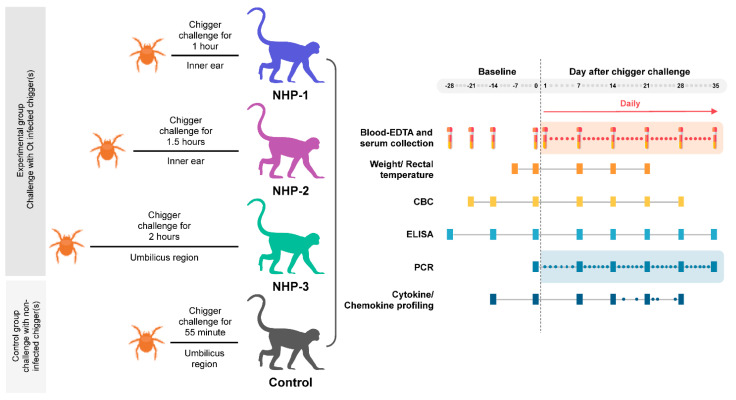
Experimental design for *Orientia tsutsugamushi* infected chigger challenged study.

**Figure 2:**
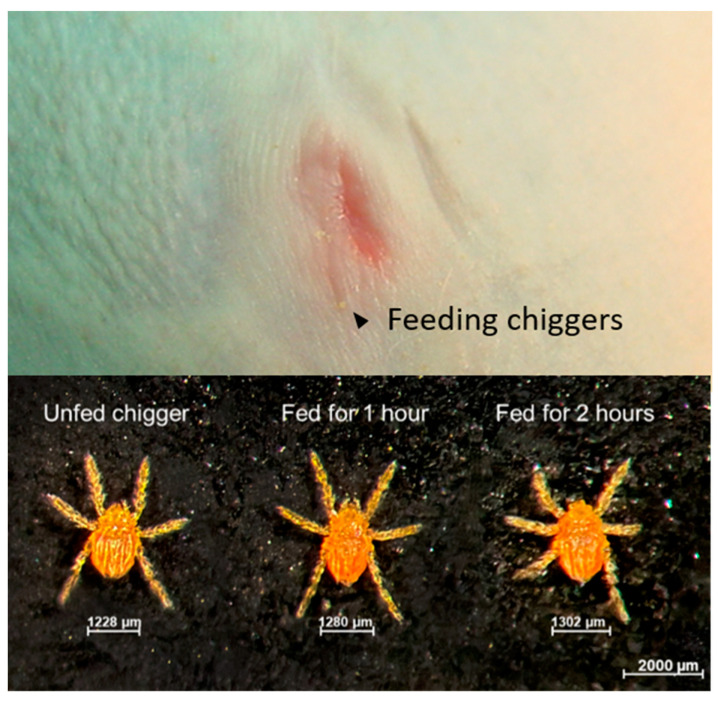
Chiggers feeding on the right umbilicus area of rhesus macaque NHP-3 is indicated using black arrows.

Three immunocompetent rhesus macaques were challenged with individual infected *O. tsutsugamushi Leptotrombidium chiangraiensis* chigger at different locations and designed time-points to determine the minimum challenging duration and optimum feeding site. The infected chiggers were selected from the naturally infected *L. chiangraiensis* (Tanskul and Linthicum) chigger colony. This bacterial strain (a member of the Karp genogroup) refer to as strain *L. chiangraiensis* line 1 (Lc-1), was not genetically modified, and thus, represented an actual strain that might cause scrub typhus infection in humans. All experiments with live infected chiggers were conducted under animal biosafety level 3 (ABSL-3) laboratory. Each rhesus macaque was challenged with individual chigger at the designed time points: NHP-1, NHP-2, and NHP-3 for 1, 1.5, and 2 h, respectively (Figure 1 and Figure 2 and Table 1). Recently, information on the optimum site for chigger challenge in the rhesus macaque model remains unknown. Our pilot study chose two locations to determine the possibility of pathogen transmission within the given time point. The selected areas included (i) the inner ear of NHP-1 and NHP-2 to mimic chigger infestation observed in wild animals and represent the location that chigger feeding was not disturbed or removed by the host and (ii) the right side of the umbilicus of NHP-3 to mimic the location where patients usually get bitten, and eschar lesions are frequently observed. As a control, a rhesus macaque was challenged with an uninfected chigger for 1 h at the inner ear site. We observed that all chiggers were firmly attached to hosts within the first 5 min post chigger placement and continued until the end of the given feeding time period.

After the chiggers were allowed to continuously feed on rhesus macaques for the designed time-points, the chiggers were carefully removed from the hosts and kept in separate rearing vials. As shown in Figure 2, partially fed chiggers were slightly enlarged, but none became fully engorged. Chiggers rapidly detached from pieces of host skin and were actively mobile within few minutes. Because of the partial feeding condition, chiggers took insufficient nutrients and did not successfully molt to the nymphal stage. Detection of *O. tsutsugamushi* in the partially engorged chiggers together with the representative from the same chigger line using pathogen-specific nested polymerase chain reaction amplification (PCR) showed that the prevalence of *O. tsutsugamushi* infection in the chigger colony was 85%. Phylogenetic analysis of the 56 kDa type-specific antigen encoding gene (*56tsa*) sequences amplified from chiggers is shown in Figure S1. *O. tsutsugamushi* DNA was detected in all chiggers except for the chiggers retrieved from NHP-2. Thus, the negative result observed for NHP-2 was because of challenge from an uninfected chigger. The results of blood chemistry and pathogen detection for this animal were excluded from all subsequent analyses.

### 2.2. Bacteremia in Infected Rhesus Macaques Showed Comparable Trend to Those Observed in Scrub Typhus Patients

Rhesus macaques bitten by chiggers harboring a highly pathogenic *O. tsutsugamushi* strain Lc-1 did not develop eschar at the bite site. Daily physical examination with assessment of skin inflammation, weight change, and rectal temperature showed that these parameters in all rhesus macaques remained within the detection limits throughout the experiment. Interestingly, NHP-1 was challenged with infected chigger for 1 h at the inner ear site presented with mild illness, moderately reduced appetite, transient anorexia, and 10% decreased body weight at the acute stage of infection starting from day 7. These signs persisted until day 28 post challenge. This macaque showed a transient increase in rectal temperature, with the highest observed temperature of 103.1 °F (39.5 °C). However, there was no association between the evaluated rectal temperature and kinetic of bacteremia in the acute phase of infection. NHP-3, which was bitten at the umbilicus area for 2 h showed moderately reduced appetite and had transient mild diarrhea observed on day 8 until day 28 post chigger challenge. No change of rectal temperature was observed.

**Figure 3:**
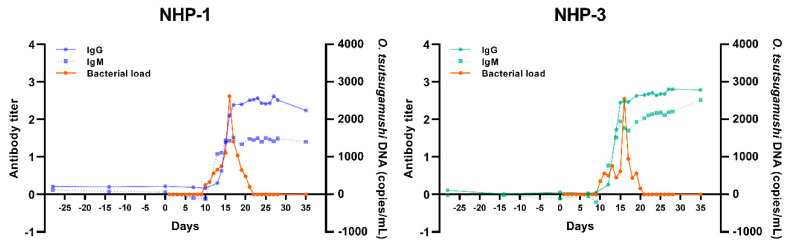

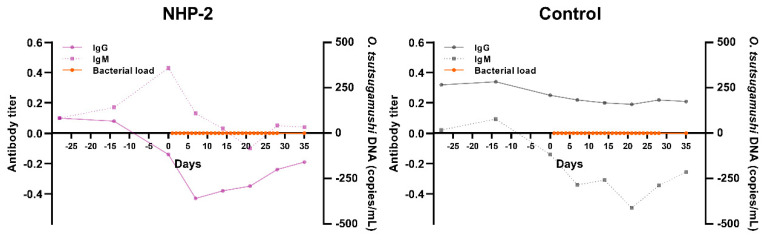
Kinetic of *O. tsutsugamushi* load in blood samples of experimental animals and specific antibody response induced by *O. tsutsugamushi* infected chigger challenge.

To further confirm the status of *O. tsutsugamushi* infection in rhesus macaques and to better understand hematogenous dissemination to distant targets, quantity and kinetic of *O. tsutsugamushi* in blood at different time-points were determined using quantitative real-time PCR (qPCR) assay targeting the conserved 47 kDa high-temperature requirement A gene (*htrA*). Bacteremia in NHP-1 and NHP-3 was first detected on day 10 post chigger challenge. Bacterial loads in the blood of NHP-1 rose from 250 copies/mL on day 10 to a peak of 2,620 copies/mL at day 16 post challenge, thereafter declining to baseline levels by day 21 post chigger challenge (Figure 3). Bacterial loads in NHP-3 rose from 350 copies/mL to 2,550 copies/mL on day 16, followed by a gradual decline with values dipping below detectable levels by day 21 post chigger challenge. The trends of bacterial DNA kinetics of NHP-1 and NHP-3 were relatively similar, except for a transient drop observed in NHP-3 on days 12, 14, and 18 post challenge. Bacterial loads peaked on the same day in both animals and were completely absent by day 21 post challenge. The *56tsa* amplicons of positive blood samples showed 100% nucleotide similarity to the *56tsa* amplified from the infected chiggers (data not shown).

### 2.3. Specific Cellular Immune Responses to O. tsutsugamushi Infection

**Figure 4:**
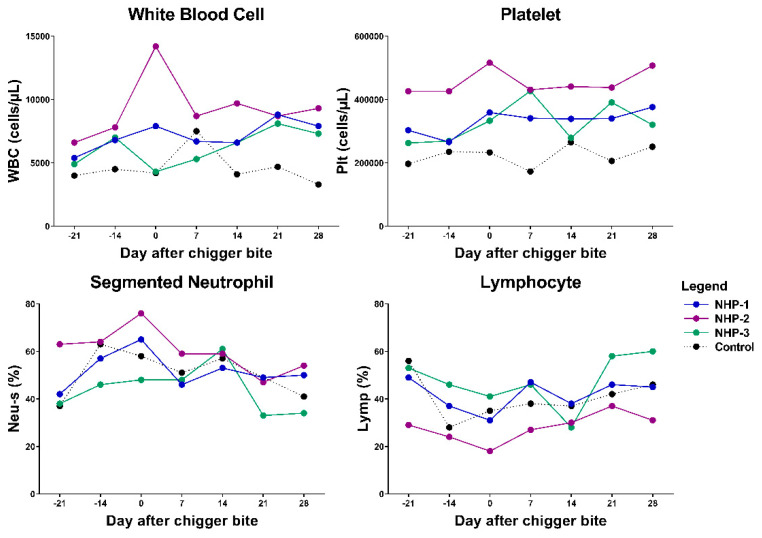
Analysis of the levels of white blood cell (WBC-leukocyte), platelet (Plt), neutrophil (Neu-s), and lymphocyte (Lymp) in blood samples from *O. tsutsugamushi* infected animals and control. Differences between groups and time points were determined using one-way ANOVA and post hoc analysis using Tukey’s multiple comparisons test. NHP-1 and NHP-3 were challenged by *O. tsutsugamushi* infected chiggers, while NHP-2 was challenged by PCR-confirmed negative chigger.

Hematological analysis revealed that the total blood cell counts of NHP-1 and NHP-3 were consistently normal with transient alterations of lymphocyte number, lymphocyte percentage, platelet number, and neutrophil percentage (Figure 4 and Figure S2 and Table S1). During the acute phase of infection on day 14 post chigger challenge, mild leukopenia, thrombocytopenia, and lymphopenia were detected in both infected rhesus macaques. Compared with the control, NHP-1 showed elevated red blood cells, hemoglobin, and mean corpuscular hemoglobin concentration. Lower levels of mean corpuscular hemoglobin and mean corpuscular volume were observed in both NHP-1 and NHP-3. Blood cells of both experimental rhesus macaques returned to normal levels by day 28 post chigger challenge.

Serum samples from experimental and control rhesus macaques were assayed for IgM and IgG using *O. tsutsugamushi* whole cell antigen enzyme-linked immunosorbent assay (ELISA). As shown in Figure 3, primary antibody responses to *O. tsutsugamushi* were observed in both NHP-1 and NHP-3, but not in NHP-2 (uninfected) and Control NHP. Seroconversion of anti-*O. tsutsugamushi* IgM and IgG were first detected in NHP-1 on days 13 and 14 post chigger challenge, while IgM and IgG were first detected in NHP-3 on day 12 post chigger challenge. The increased IgM and IgG antibody levels of NHP-3 were sustained until day 38. Although NHP-3 had a mild symptomatic infection, the host’s cellular and adaptive immunity were observed with similar kinetics, as seen in the NHP-1 symptomatic infection. The time to detection of initial IgM and IgG responses in both infected animals was similar. These results indicated that clinical manifestations were not correlated with the host’s immune response. NHP-2 was challenged with uninfected chigger and neither this macaque, nor the control animal, developed a productive infection or *O. tsutsugamushi*-specific antibody responses.

### 2.4. Elevated Levels of Serum Cytokines and Chemokines Are Associated with Progressive O. tsutsugamushi Infection

**Figure 5:**
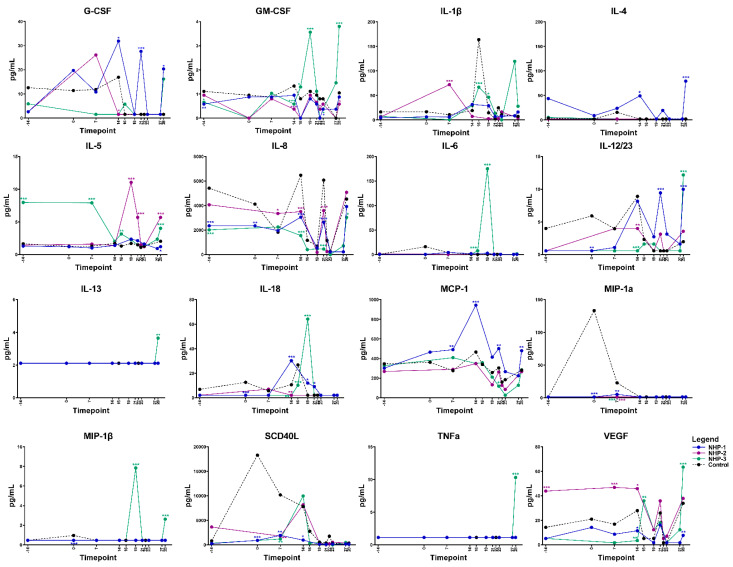
Selected cytokine/chemokine levels in rhesus macaques during the progress of *O. tsutsugamushi* infection via chigger bite. * *p* < 0.05, ** *p* < 0.01, *** *p* < 0.001.

**Figure 6:**
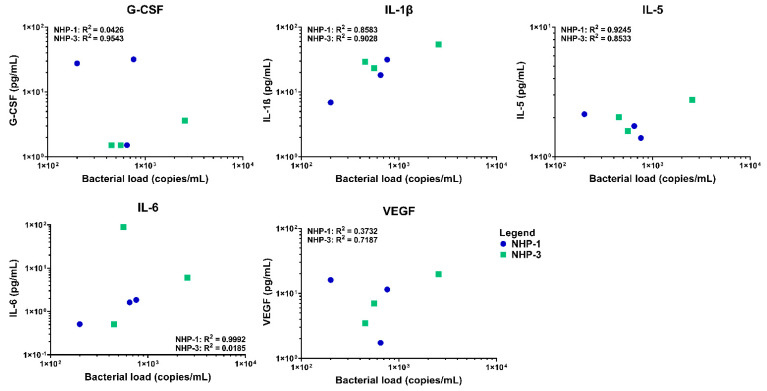
Correlation between G-CSF, IL-1β, IL-5, IL-6, and VEGF production and load *O. tsutsugamushi* infection in blood samples of experimental rhesus macaques. The linear regression is shown in each graph. Correlation efficiency and P value were calculated based on the duplication of each individual samples.

Elevations in serum cytokine and chemokine levels were observed in both infected rhesus macaques NHP-1 and NHP-3 over time using multiplex cytokine/chemokine analysis. Overall, NHP-1 and NHP-3 challenged with the infected chigger showed evidence of stronger T helper cell type 1 (Th1) responses, although mixed Th1 and Th2 responses were also observed. On day 7 post chigger challenge, levels of most cytokines/chemokines analyzed remained relatively close to the baseline level except for G-CSF, IL-1β, MCP-1, MIP-1α/CCL3, and TGF-α. Analysis of proinflammatory cytokine/chemokine profiles during the acute phase of infection between days 14 and 16 post challenge showed marked increases of G-CSF, GM-CSF, IL-1β, IL-4, IL-8, IL-12/23 (p40), IL-18, MCP-1/CCL2, SCD40L, TGF-α, and VEGF (Figure 5 and Figure S3). Multivariable linear regression analyses were conducted to assess correlations between levels of proinflammatory cytokine/chemokines and concentrations of *O. tsutsugamushi* in blood. Acute phase elevation of G-CSF, IL-1β, IL-5, IL-6, and VEGF levels showed a positive association with increasing *O. tsutsugamushi* loads in the blood of NHP-1 and NHP-3 (Figure 6 and Figure S4). At the convalescent phase from day 21 to day 28, transient alterations in levels of G-CSF, GM-CSF, IL-2, IL-1β, IL-4, IL-5, IL-8, IL-12/23, IL-13, MCP-1, MIP-1β, TGF-α, TNF-α, and VEGF were observed. Comparing levels of cytokines at the acute and convalescent phases, we found that concentrations of IL-1ra, IL-6, IL-18, and MCP-1 were relatively higher during the acute phase of infection, suggesting that these cytokines potentially play key roles in controlling *O. tsutsugamushi* infection. Concentrations of two cytokines, IL-5 and IL-8, decreased in either infected rhesus macaques compared to the control or NHP-2 (uninfected) throughout the infection. No differences in levels of IFN-γ and IL-17α were observed between *O. tsutsugamushi*-infected rhesus macaques and control or between the acute and convalescent phases.

## 3. Discussion

The present study showed that a single chigger was capable of transmitting *O. tsutsugamushi* pathogens and cause infection in rhesus macaque. The minimum duration that chiggers require to form a functional stylostome and transmit the pathogen was as short as 1 h after attachment on the host, implying that the risk of scrub typhus infection is high despite chigger removal within a short period of time after biting. In fact, chigger bite is painless and hard to notice, due to the microscopic size of the vector. This minimum attachment time was unexpectedly shorter than the time required for pathogen transmission by other acari-associated taxa, including ticks [46,47]. To date, the mechanisms involved in early and rapid transmission of *O. tsutsugamushi* within a short timeframe after attachment are incompletely understood. Part of the explanation may be that *O. tsutsugamushi* systemically infects chiggers, resulting in high bacterial loads in the secretory granules within the cytoplasm of salivary gland cells [48,49,50]. Once chigger started to feed and retrieve nutrition, *O. tsutsugamushi* cells are stimulated and maturated into an active form and release within salivary secretions. Similar findings were observed in the murine model that the chigger stylostomes were well-formed and penetrated the epidermis or dermis of host skin after 24 h of attachment [44]. Additionally, recent studies suggested that different chigger species had distinctive characteristics in terms of the depth and width of the stylostome [44,51,52]. These different forms of the stylostome are believed to have direct effects on the degree of dermal inflammation and epidermal hyperplasia in murine models. However, the factors impacting the formation of distinctive patterns of stylostomes remain to be elucidated. It is also possible that components in chigger salivary complexes could potentially modulate the host’s defense mechanism to promote pathogen infection and dissemination, as well as to enhance the completion of the chigger feeding process. Comprehensive studies of the protein composition in salivary complexes or salivary gland extracts from actively feeding chiggers and maturation of *O. tsutsugamushi* in the vector are being studied to better understand the factors that promote rapid feeding and pathogen transmission from chigger to host, as well as correlations between stylostome formation, transmission efficiency, and disease severity.

The results from our pilot study suggested that either the inner ear or the umbilicus area could be considered optimum sites for chigger challenge to develop rhesus macaque model of scrub typhus. Although the experimental rhesus macaques were challenged with infected chiggers in different locations and times, both NHP-1 and NHP-3 developed a symptomatic or mild symptomatic infection and showed relatively similar trends of cellular and humoral immune responses. The experimental rhesus macaques showed bacteremia and IgM and IgG seroconversion. Detectable bacterial loads were observed as early as day 10 after chigger challenge and peaked at day 16 before declining to undetectable levels on day 21. In our disease model, the kinetics of bacteria circulation in the blood and the relapsing pattern of bacteremia after the generation of specific humoral immune responses were remarkably similar to those seen in scrub typhus patients [53]. Additionally, following *O. tsutsugamushi* infection, rhesus macaques had significant changes in levels of proinflammatory cytokines and chemokines. Levels of G-CSF, IL-1β, IL-5, and IL-6, and chemoattractant VEGF were significantly altered during the acute phase of infection with concomitant detection of bacterial loads in the blood. These cytokines and chemokines were likely directly involved in the inflammatory process induced by infection with *O. tsutsugamushi*.

One of the important findings of our pilot study was that rhesus macaques infected with *O. tsutsugamushi* developed nonlethal, mild infections. This finding contrasted with the previous observations in murine models showing that the Lc-1 strain caused highly pathogenic and lethal infection [45,54]. Several factors may be responsible for this difference, including different inoculation doses of chiggers during the short transmission period vs. the lethal dose used in other experiments, variation in the pathogenicity of bacteria itself, and differences in host susceptibility. In future NHP scrub typhus infection model development, chigger mites should be allowed to feed for longer times or feed ad libitum until fully engorged, increase the number of infected chiggers to at least two or three from the mite colony in which 85% infected, to ensure that one is infected to transmit the pathogen, which will allow us to better understand the impact of complete stylostome formation on host skin reaction, the effect of chigger saliva and dose of bacterial inoculum from chigger on disease progression and pathogenesis. Further histological analysis of skin at the chigger bite site would enhance our understanding of the stylostome characteristics of Leptotrombidium chiggers.

While we believe that data from our pilot study provide fundamental information for further comprehensively characterize host immune response to scrub typhus infection, we also recognized limitations in our study. In the initial phase of the disease model development, only a small number of animals were used. This consequently limited statistical confidence or comparison of the recent findings with previously reported models. To determine the suitable chigger challenge condition with a very limited number of animals per group, different feeding time-points and challenge locations were used between control and experimental animals, making direct comparisons between control and experimental groups difficult. Additionally, even though bacterial loads in blood samples were quantitated using fresh specimens, proinflammatory cytokine/chemokine profiling was performed using stored serum samples. Long term storage of specimens (more than 13 years at −80 °C) might cause degradation of important analytes and change the results. The unavailability of fresh blood samples limits the comprehensive investigation of a specific host’s cellular response to the infection. Although our model remains preliminary, we believe it could be highly useful for other researchers working in developing the chigger challenge NHP model and could catalyze rapid progress to improve our understanding of the disease.

## 4. Materials and Methods

### 4.1. Nonhuman Primates

The experiments described herein were approved by the Armed Forces Research Institute of Medical Sciences (AFRIMS) Institutional Animal Care and Use Committee (IACUC) under protocol number 07-04 “Evaluation of scrub typhus vaccine candidates by intradermal injection with *Orientia tsutsugamushi* and by natural feeding of scrub typhus-infected chiggers.” Animal studies were performed in accordance with the guidelines of the Public Health Service Policy on Humane Care and Use of Laboratory Animals, the Guide for the Care and Use of Laboratory Animals 8th edition, the Animal Welfare Act, and all applicable U.S. Department of Agriculture, Office of Laboratory Animal Welfare and U.S. Department of Defense guidelines. During this study, animals were housed inside an ABSL-3 facility. All procedures were performed at AFRIMS Bangkok, Thailand, an AAALAC International-accredited facility.

Four immunocompetent adult Indian-origin rhesus macaques were randomly allocated to each experimental arm, regardless of sex or age (Table 1). Rhesus macaques were negative for preexisting pathogen-specific antibodies against *O. tsutsugamushi*, simian immunodeficiency virus, simian retrovirus, simian T-lymphotropic virus, and macacine herpesvirus. All animals received standard primate feed, fresh fruit daily, and had free access to water following regulations and the environmental enrichment program as previously described [36]. Complete physical examinations were performed by AFRIMS laboratory animal veterinarians prior to the experiment. Rectal temperatures and weights were taken prior to each procedure by qualified veterinary technicians.

### 4.2. Leptotrombidium sp. Chigger Mite Colonies

*O. tsutsugamushi* infected *L. chiangraiensis* (Tanskul and Linthicum) chiggers originally collected from wild rodents in endemic areas of Chiang Rai province in northern Thailand and maintained under ABSL-3 facility, were used for the experiment [55]. This chigger line was chosen, due to high pathogenicity and nearly 100% mortality rate in murine models infected with *O. tsutsugamushi* strain Lc-1 either through direct chigger transmission or intraperitoneal inoculation of the infected tissue homogenates [45,54,56,57]. Genetic characterization of the *O. tsutsugamushi* strain in the *L. chiangraiensis* colony, *O. tsutsugamushi* strain Lc-1, suggested the high and consistent infection of the Karp-genogroup strain sharing 90% nucleotide identity in the *56tsa* gene fragment with the Karp prototype (GenBank accession EF213092) [57].

During chigger colony maintenance, we observed that *L. chiangraiensis* chiggers aged between 3 and 4 weeks old were active feeders and usually required only a few minutes to become rigidly attached to host skin. Chiggers at this age originated from the same parental line, and generations were used throughout the experiment to control infection consistency between different generations. Pathogen-free *L. chiangraiensis* chigger colonies, housed separately in a BSL-1 laboratory at the Department of Entomology, AFRIMS, were produced using similar procedures and used as negative controls. The infection status of the control group chiggers was confirmed by testing for *O. tsutsugamushi* infection using specific PCR of the *56tsa* together with mouse passage experiments [58].

### 4.3. Chigger Challenge of Rhesus Macaques

#### 4.3.1. Chigger Placement Experiment

Prior to the experiment, all rhesus macaques underwent complete physical examination, and 1.5 mL of whole blood (0.5 mL each into ethylenediaminetetraacetic acid (EDTA), heparinized, and serum separator tubes) was collected on days −28, −21, −14, and on the day of the experiment (day 0) immediately prior to chigger challenge. Complete blood counts, blood chemistry, and serological analyses were performed. The pole-collar-chair restraint procedure was used to avoid unnecessary anesthesia during brief procedures for blood collection. On the day of the experiment (day 0), all experimental animals were anesthetized by intramuscular injection of a mixture of ketamine (5–20 mg/kg) and xylazine (1–2 mg/kg). The abdominal fur was clipped, and skin was sanitized with 70% isopropyl alcohol and air-dried for 10 min for odor elimination. Chiggers were placed on different areas of the body and allow to feed for different periods to identify appropriate sites and attachment time (Table 1 and Figure 1). Chigger placement in the inner ear was monitored closely using a headband magnifier to ensure complete attachment, while chigger feeding on the area near the umbilicus was controlled by drops of distilled water and a rubber band to control the feeding area. Chigger feeding experiments were performed under close monitoring by laboratory technical staffs to ensure that chiggers were firmly attached and to avoid loss of *O. tsutsugamushi* infected chiggers. At designed time points, chiggers were carefully retrieved from hosts using fine forceps and kept in separate rearing vials to allow them to molt into nymphal and adult stages. Chiggers that did not successfully develop into nymphs were stored in 70% ethyl alcohol at 4 °C for confirmation of *O. tsutsugamushi* infection.

#### 4.3.2. Clinical Observation and Blood Sample Collection

After chigger feeding, animal health and clinical signs were observed by AFRIMS veterinarians, three times daily until day 38 post chigger challenge. Animals were monitored for decreased activity, rectal temperature fluctuation, and food consumption. Fever condition was considered if the animal had rectal temperature increased more than 2 Fahrenheit units from the baseline level or higher than 103.1 °F (39.5 °C).

Blood (1.5 mL) was drawn from the femoral vein on day 21 and day 14 prior to the challenge and thereafter at 7-day intervals until day 28 post chigger challenge and were analyzed for complete blood count and blood biochemistry. Serum samples were collected on day 7, day 9, daily between day 10 and day 28, and again on day 38 post chigger challenge were analyzed for serum biochemistry, proinflammatory cytokine/chemokine levels, and specific antibodies against *O. tsutsugamushi*. To study the kinetics of bacterial replication, daily whole blood specimens were collected from rhesus macaques and used for pathogen quantification by *O. tsutsugamushi*-specific qPCR assay [59].

### 4.4. Quantitation of O. tsutsugamushi Concentrations in Blood and Chigger Samples

Genomic DNA was extracted from 100 µL of blood-EDTA samples and the unengorged chiggers using a QIAamp DNA mini kit (Qiagen, Hilden, Germany) following the manufacturer’s recommendations with some modifications for chigger samples as previously described [6]. The extracted DNA of blood samples or chiggers were eluted using 50 µL or 35 µL of AE elution buffer (Qiagen), respectively. *L. chiangraiensis* chiggers from the infected colony and an isolate of *O. tsutsugamushi* strain Karp were used as positive controls. Non-template controls, including a reagent control and distilled water, were included in all DNA extractions to eliminate the possibility of cross-contamination between assays. The presence of *O. tsutsugamushi* in monkey blood samples was assessed using qPCR specific for the *htrA* gene following the previously described protocol [59]. The qPCR efficiency was 100%, and the limit of detection was 3 copies/µL of monkey bloods. The bacterial load in the blood sample was expressed as a genomic equivalent copy of htrA gene/µL or mL. Copy number is calculated based on the usage of 5 µL of a total of 50 µL of extracted DNA in each qPCR reaction. To sequence *O. tsutsugamushi* positive samples, a 650 nucleotide fragment of *56tsa* was amplified following the previously described protocol [60]. Nucleotide sequences of the *56tsa* were aligned with reference *Orientia* sequences using the ClustalW algorithm in the BioEdit program. Neighbor-joining and maximum likelihood phylogenetic trees were constructed using MEGA software (www.megasoftware.net/home).

### 4.5. Assessment of Host Immune Responses to O. tsutsugamushi Infection

#### 4.5.1. Hematological Analysis

Blood EDTA samples were analyzed for 24 hematological parameters using an automatic hematological analyzer (K-800, Sysmex, Japan). The hematological parameters include the number of white blood cells (WBC), number of red blood cells (RBC), hematocrit (HCT), hemoglobin (HGB), platelets (PLT), neutrophil percentage (NEUT%), neutrophil (NEUT#), lymphocyte percentage (LYMPH%), lymphocyte (LYMPH#), monocyte percentage (MONO%), monocyte (MONO#), eosinophil percentage (EO%), eosinophil (EO#), basophilic leukocyte percentage (BASO%), basophil (BASO#), mean corpuscular hemoglobin concentration (MCHC), mean corpuscular hemoglobin (MCH), mean corpuscular volume (MCV), mean platelet volume (MPV), plateletcrit (PCT), red blood cell volume distribution width-SD (RDW-SD), red blood cell volume distribution width-CV (RDW-CV), plate volume distribution width (PDW), and platelet large cell ratio (P-LCR).

#### 4.5.2. Serological Analysis

Serum samples collected at designed time points were analyzed for specific IgM and IgG against *O. tsutsugamushi* using ELISA. ELISA was performed by using whole cell antigen of Karp strain (Papua New Guinea) of *O. tsutsugamushi* provided by the Viral and Rickettsial Diseases Department of the Naval Medical Research Center, Silver Spring, MD, following previously described protocol [61]. Additionally, serum samples from the AFRIMS specific pathogen-free rhesus macaque colony and positive sera from patients with confirmed scrub typhus infection were used as negative and positive controls, respectively. All sera were diluted to 1:50 in duplicate to evaluate serostatus. The mean optical densities (ODs) of tested sera were calculated after subtracting the ODs in wells without antigen from the ODs in wells with antigen. The cut-off values for IgM or IgG were calculated based on the values of negative control samples from rhesus macaques collected prior to the chigger challenge and from healthy rhesus macaques. A sample was considered positive at a given dilution if the mean of blank-subtracted ODs was greater than the mean OD of negative controls plus three standard deviations. The antibody values were determined to be the inverse of the highest dilution with a net absorbance of 0.200 or greater. Initially, serum samples of all macaques at days −28, −14, 0, 7, 14, 21, and 28 post chigger challenge were assayed for the presence of *Orientia*-specific IgM and IgG. In any rhesus macaques showing seropositivity, additional serum samples collected at other time points (days 9 and 12, between days 15–28, and day 35) were analyzed to assess the kinetics of antibody responses following infection.

#### 4.5.3. Multiplex Serum Cytokine and Chemokine Assay

Serum samples (undiluted) from all rhesus macaques were assessed for the presence of proinflammatory cytokine/chemokine levels using a 23-plex Milliplex MAP nonhuman primate cytokine magnetic bead panel-immunology multiplex assay (EMD Millipore, Billerica, MA, USA). The analytes include granulocyte-colony stimulating factor (G-CSF), granulocyte macrophage-colony stimulating factor (GM-CSF), interferon (IFN)-γ, interleukin (IL)-10, IL-12/23 (p40), IL-13, IL-15, IL-17, IL-18, IL-1 receptor antagonist (IL-1ra), IL-1β, IL-2, IL-4, IL-5, IL-6, IL-8, monocyte chemotactic protein-1 (MCP-1), macrophage inflammatory protein (MIP)-1α, MIP-1β, transforming growth factor (TGF)-α, tumor necrosis factor (TNF), vascular endothelial growth factor (VEGF), and a soluble cluster of differentiation 40 ligand (sCD40L). Each serum sample was assayed in duplicate without dilution. The experiment was performed along with duplicates of seven dilutions of cytokine standards and a low and high concentration quality control sample provided by the manufacturer. Multianalyte profiling was performed using a Luminex-100 system, and data were analyzed using Bio-Plex Manager software (Bio-Rad Laboratories, Hercules, CA, USA). Cytokine/chemokine concentrations were presented as average values of duplicate tests. Samples with analyte concentrations below the detection limit were assigned values of 0 and were retained in the analysis.

### 4.6. Data Analysis

All data were presented as means plus or minus standard deviation. GraphPad Prism version 8 (San Diego, CA, USA) was used for data analysis. Increased expression of cytokines/chemokines after infection was assessed by establishing a threshold of reactivity.

## 5. Conclusions

Scrub typhus research has historically been hampered by the lack of reliable and comparable disease models. Better models that more faithfully mimic the process of infection in humans are necessary to understand the disease, and hopefully, to test vaccine efficacy. In the present pilot study, we developed a chigger transmission-based scrub typhus disease model using rhesus macaques. Using *L. chiangraiensis* chigger colonies infected with *O. tsutsugamushi* strain Lc-1, we developed a chigger challenge method of rhesus macaques that faithfully mimics the natural route of pathogen transmission. We investigated that the minimal time that chigger requires to successfully transmit *O. tsutsugamushi* to rhesus macaques was as rapid as 1 h after attachment on host skin. *O. tsutsugamushi* infection in rhesus macaques via chigger challenge recapitulated the time of disease onset, bacteremia, and cellular and humoral immunities observed in patients, strengthening the value of this disease model.

## Figures and Tables

**Table 1:** Clinical presentation of rhesus macaques after chigger mite feeding.

Animal ID	Sex	Location for Chigger Placement	Age (Years)	Sign of Illness	ChiggerFeeding Time	Physical Condition	CBC	Serum Chemistry	Bacteremia
NHP-1	Female	Inner ear	16	Moderate reduced appetite, transient anorexia, and loss weight by 10% on day 7	1 h	Normal	Normal	Normal	Yes
NHP-2	Male	Inner ear	16	Normal	1.5 h	Normal	Normal	Normal	No
NHP-3	Male	Umbilicus	15	Moderate reduced appetite and transient mild diarrhea starting from day 8 until day 28	2 h	Normal	Normal	Normal	Yes
Control	Female	Inner ear	15	Normal	1 h	Normal	Normal	Normal	No

The observation was performed on study days 0, 7, 14, 27, and 28 post chigger challenge. Normal = rhesus macaque has normal activity and appetite. The monkey ate more than 21 monkey biscuits per day or more than 11 biscuits in the afternoon. Moderate reduced appetite = monkey ate 6 to 14 biscuits per day or 4 to 7 biscuits in the afternoon. Anorexia = the monkey ate less than 5 pieces biscuits per day or less than 3 biscuits in the afternoon.

## Data Availability

The data of this manuscript is available upon request to corresponding author.

## Supplementary Materials

The following are available online at https://www.mdpi.com/article/10.3390/pathogens10081028/s1, Table S1. Complete blood count of rhesus macaques, Figure S1. Phylogenetic analysis of *O. tsutsugamushi* in *Leptotrombidium chaingraiensis* chiggers from the colonies, Figure S2. Hematological analysis of rhesus macaques after *O. tsutsugamushi* infection or non-infected chigger bite (control), Figure S3. Serum levels of 23 cytokine/ chemokine in rhesus macaques during *O. tsutsugamushi* infection via chigger bite, Figure S4. Immunological correlation analysis of serum cytokine/ chemokine of all macaques and bacteremia during acute phase of infection.

## Disclaimer

Material has been reviewed by the Walter Reed Army Institute of Research. There is no objection to its presentation and/or publication. The opinions or assertions contained herein are the private views of the authors, and are not to be construed as official, or as reflecting true views of the Department of the Army or the Department of Defense. The research was conducted under an approved animal use protocol in an AAALAC accredited facility in compliance with the Animal Welfare Act and other federal statutes and regulations relating to animals and experiments involving animals and adhered to principles stated in the Guide for the Care and Use of Laboratory Animals, NRC Publication, 2011 edition.
