# Supplementary material for: Establishment of a Rhesus Macaque Model for Scrub Typhus Transmission: Pilot Study to Evaluate the Minimal Orientia tsutsugamushi Transmission Time by Leptotrombidium chiangraiensis Chiggers
Source: Pathogens. 2021 Aug 13;10(8):1028. doi: 10.3390/pathogens10081028 (PMC8402083; doi:10.3390/pathogens10081028)
Supplement: Supplementary file 1 [file pathogens-10-01028-s001.zip › pathogens-1279193-supplementary.pdf]

Supplement table and figure

Table S1. Complete blood count of rhesus macaques

Comparison CBC results at differential day after feeding for control group

Tests	Units	Day after feeding for control group						
		-21 day	-14 day	0 day	7 day	14 day	21 day	28 day
RBC	10^6/ul	4.79	4.76	4.83	5.01	4.93	5.12	5.02
Hct	%	38	38	39	40	39	41	40
Hb	gm%	12.0	12.2	12.2	12.7	12.3	12.7	12.5
WBC	cells/ul	3,700	4,500	4,200	7,500	4,100	4,700	3,300
Plt	cells/ul	154,000	235,000	233,000	173,000	265,000	206,000	251,000
MCV	fl	79.7	79.4	79.7	79.0	79.9	79.9	80.1
MCH	pg	25.1	25.6	25.3	25.3	24.9	24.8	24.9
MCHC	g/dl	31.4	32.3	31.7	32.1	31.2	31.1	31.1
Neu-s	%	35	63	58	51	57	49	41
N-b	%	0	2	2	2	2	3	3
Lymp	%	56	28	35	38	37	42	46
Bas	%	1	0	1	1	0	1	1
Eos	%	1	0	1	1	0	1	1
Mon	%	4	3	2	3	1	1	2

Comparison CBC results at differential day after feeding for NHP-1

Tests	Units	Day after feeding for NHP-1						
		-21 day	-14 day	0 day	7 day	14 day	21 day	28 day
RBC	10^6/ul	6.38	5.99	6.26	6.16	6.12	6.06	6.18
Hct	%	44	41	43	42	42	41	42
Hb	gm%	14.5	14.0	14.7	14.2	14.1	13.8	14.2
WBC	cells/ul	5,400	6,800	7,900	6,700	6,600	8,800	7,900
Plt	cells/ul	303,000	266,000	359,000	341,000	339,000	340,000	376,000
MCV	fl	69.1	68.6	68.1	68.8	68.3	68.2	68.1
MCH	pg	22.7	23.4	23.5	23.1	23.0▼	22.8▼	23.0▼
MCHC	g/dl	32.9	34.1	34.5	33.5	33.7▼	33.4▼	33.7
Neu-s	%	42	57	65	46	53	49▼	50▼
N-b	%	1	2	2	1	2	0	1
Lymp	%	49	37	31	47	38	46▲	45▲
Bas	%	1	0	1	0	3	1	1
Eos	%	1	0	1	0	3	1	1
Mon	%	6	3	1	3	1▼	2	2

Comparison CBC results at differential day after feeding for NHP-3

Tests	Units	Day after feeding for NHP-3						
		**-21 day**	**-14 day**	**0 day**	**7 day**	**14 day**	**21 day**	**28 day**
RBC	10^6/ul	5.35	5.33	5.43	5.58	5.44	5.09	5.43
Hct	%	40	40	41	42	41	39	41
Hb	gm%	13.0	13.4	13.2	13.4	13.0	12.3	13.0
WBC	cells/ul	4,900	7,000	4,300	5,300	6,600	8,100	7,300
Plt	cells/ul	263,000	269,000	333,000	427,000	279,000	391,000	320,000
MCV	fl	75.0	75.2	75.3	76.0	76.1	76.2	74.3
MCH	pg	24.3	25.1	24.3	24.0	23.9▼	24.2▼	24.4▼
MCHC	g/dl	32.4	33.4	32.3	31.6	31.4▼	31.7▼	32.8
Neu-s	%	38	46	48	48	61	33▼	34▼
N-b	%	4	1	2	2	5	2	0
Lymp	%	53	46	41	46	28	58▲	60▲
Bas	%	0	0	0	0	0	0	0
Eos	%	0	0	0	0	0	0	0
Mon	%	2	3	1	4	0▼	3	3

'▲' means that the- values showed significantly increased after chigger challenge. '▼' means that the values showed significantly decreased after chigger challenge.

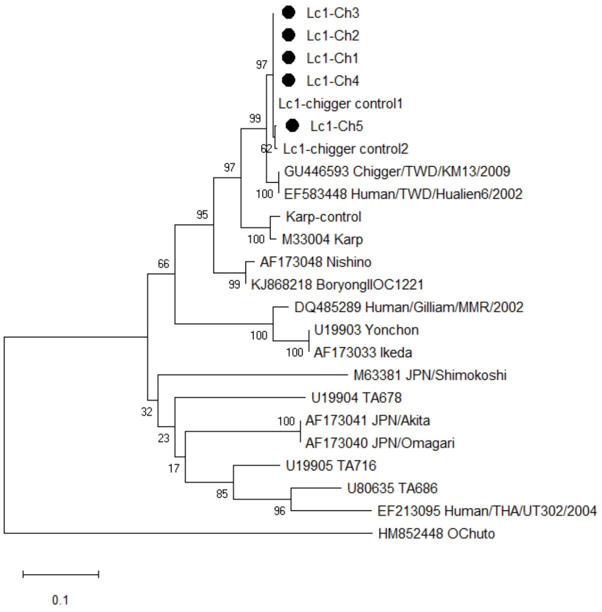

Figure S1. Phylogenetic analysis of *O. tsutsugamushi* in *Leptotrombidium chaingraiensis* chiggers from the colonies. The evolutionary tree was inferred by using Maximum likelihood method and General Time Reversible model. A discrete Gamma distribution was used to model evolutionary rate differences among sites. The tree is drawn to scale, with branch lengths measured in the number of substitutions per site. Numbers at nodes indicate bootstrap value at 1000 replications.

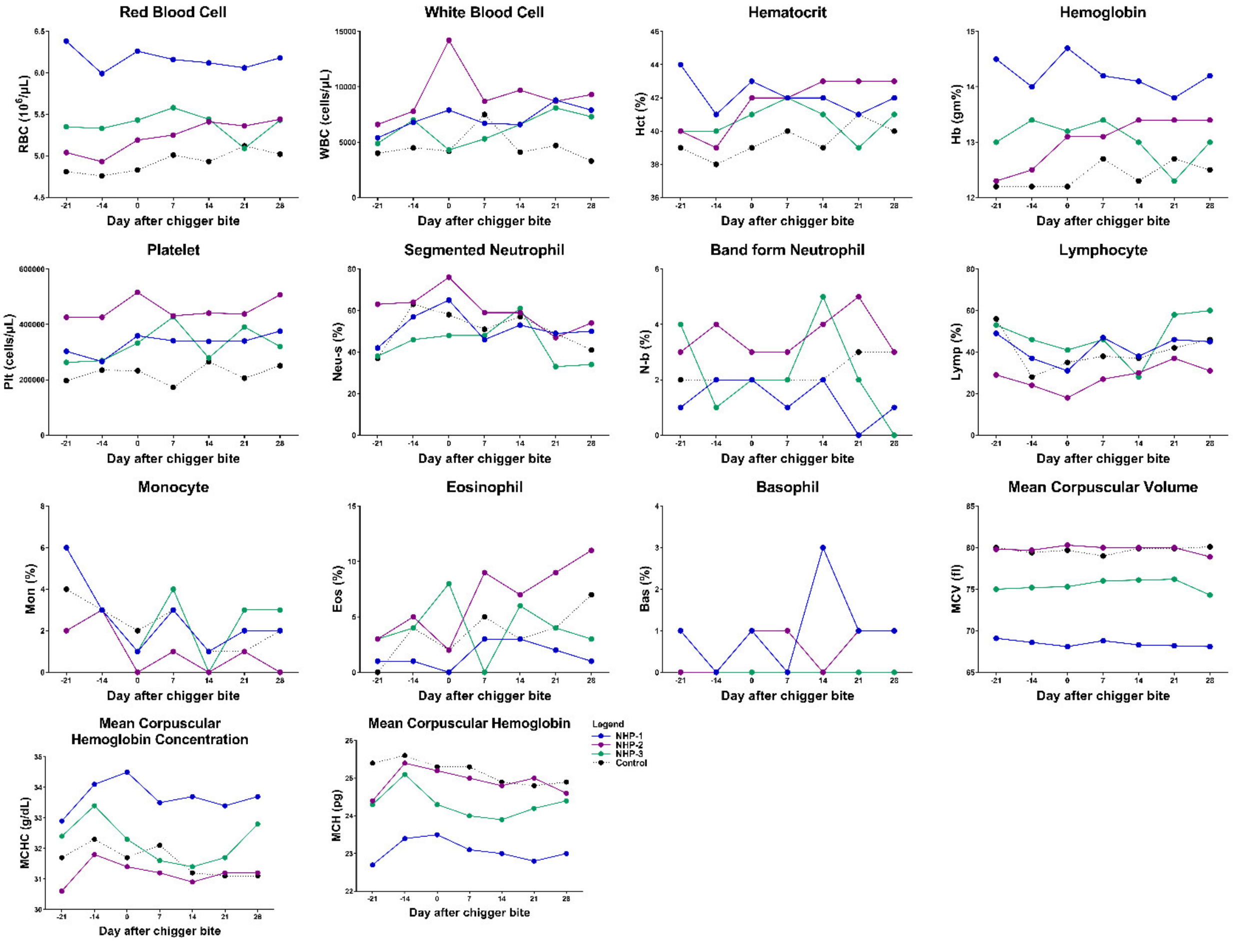

Red Blood Cell
RBC (10⁶/µL)
White Blood Cell
WBC (cells/µL)
Hematocrit
Hct (%)
Hemoglobin
Hb (gm%)
Platelet
Plt (cells/µL)
Segmented Neutrophil
Neu-s (%)
Band form Neutrophil
N-b (%)
Lymphocyte
Lymp (%)
Monocyte
Mon (%)
Eosinophil
Eos (%)
Basophil
Bas (%)
Mean Corpuscular Volume
MCV (fl)
Mean Corpuscular Hemoglobin Concentration
MCHC (g/dL)
Mean Corpuscular Hemoglobin
MCH (pg)
Day after chigger bite
Legend
NHP-1
NHP-2
NHP-3
Control

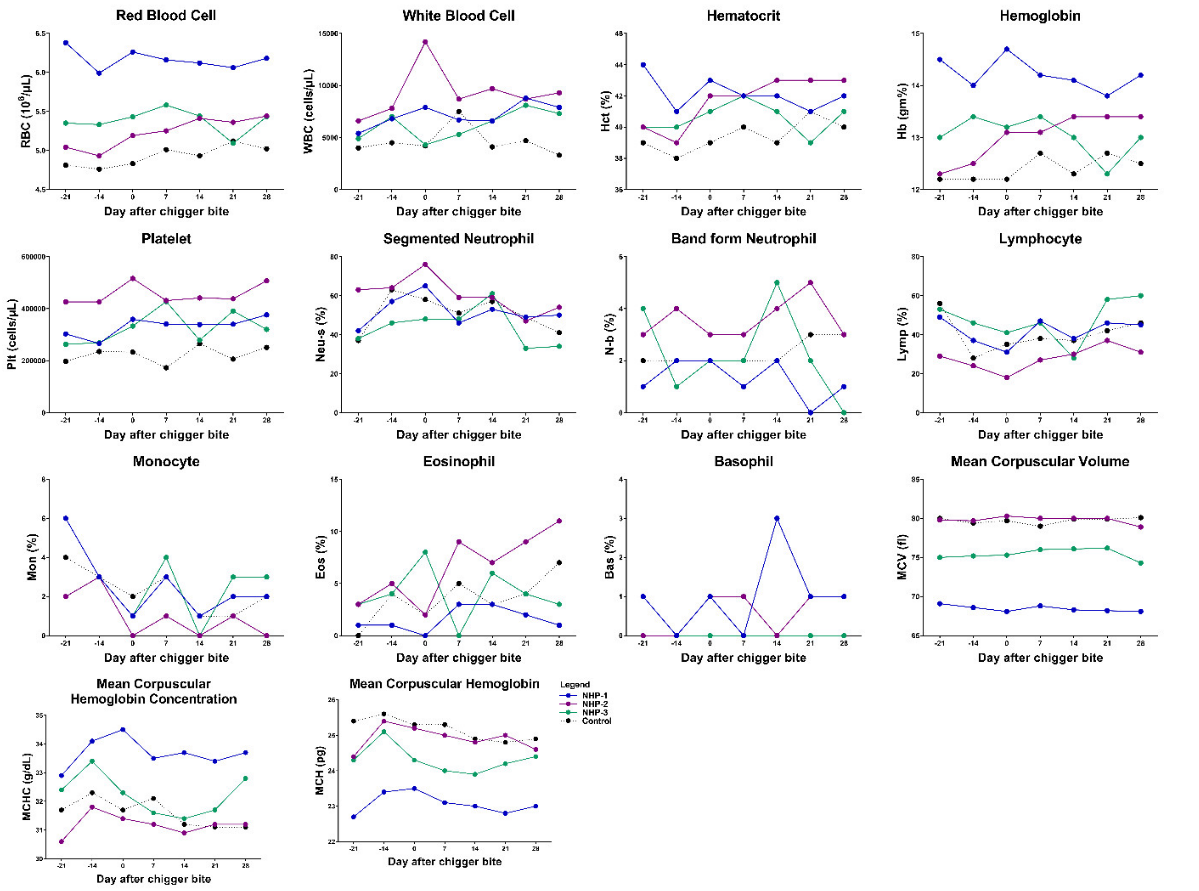

Figure S2. Hematological analysis of rhesus macaques after *O. tsutsugamushi* infection or non-infected chigger bite (control).

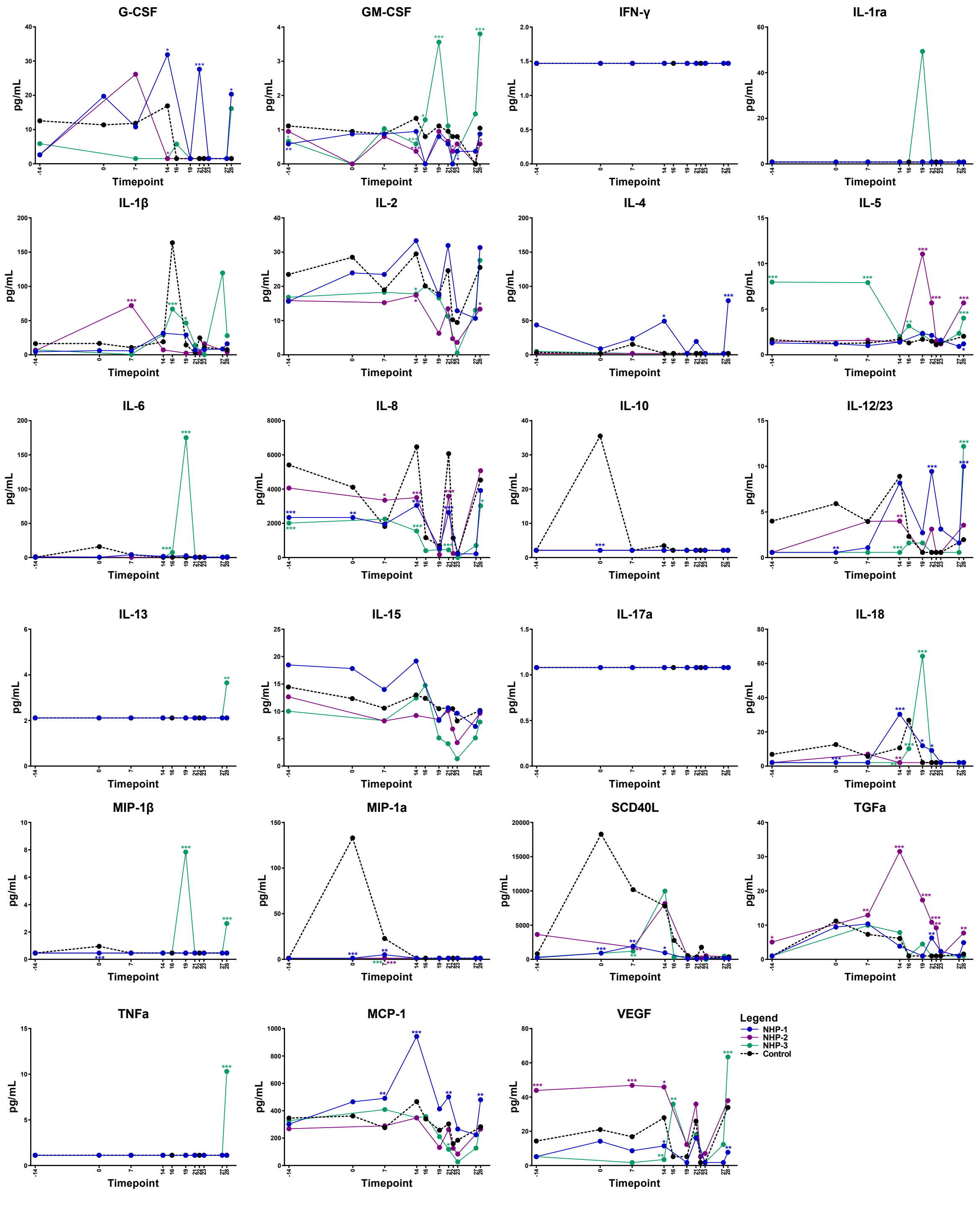

Figure S3. Serum levels of 23 cytokine/ chemokine in rhesus macaques during *O. tsutsugamushi* infection via chigger bite.

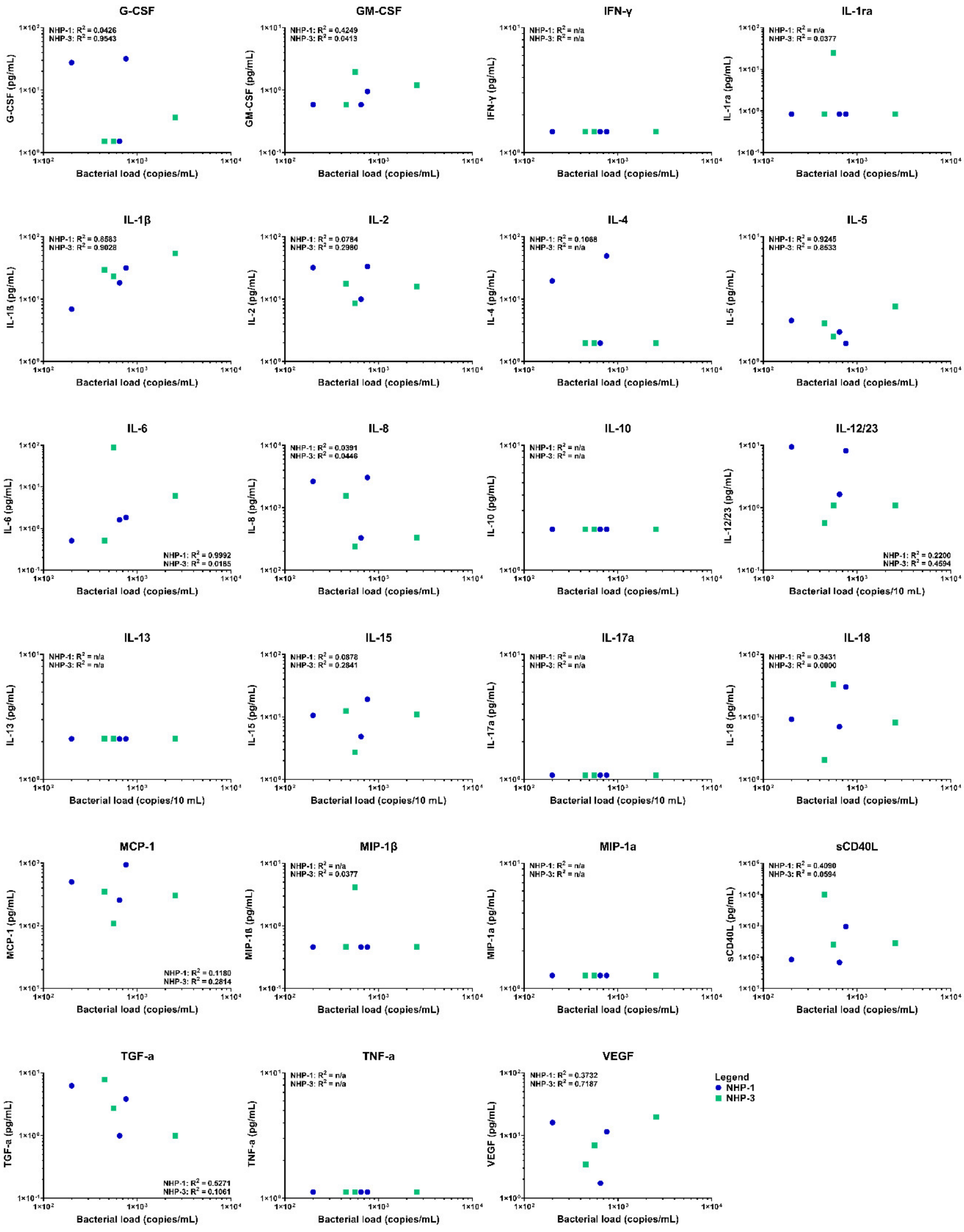
G-CSF
NHP-1: R^2 = 0.0426
NHP-3: R^2 = 0.9543
G-CSF (pg/mL)
Bacterial load (copies/mL)
GM-CSF
NHP-1: R^2 = 0.4249
NHP-3: R^2 = 0.0413
GM-CSF (pg/mL)
Bacterial load (copies/mL)
IFN-γ
NHP-1: R^2 = n/a
NHP-3: R^2 = n/a
IFN-γ (pg/mL)
Bacterial load (copies/mL)
IL-1ra
NHP-1: R^2 = n/a
NHP-3: R^2 = 0.0377
IL-1ra (pg/mL)
Bacterial load (copies/mL)
IL-1β
NHP-1: R^2 = 0.8583
NHP-3: R^2 = 0.9028
IL-1β (pg/mL)
Bacterial load (copies/mL)
IL-2
NHP-1: R^2 = 0.0784
NHP-3: R^2 = 0.2980
IL-2 (pg/mL)
Bacterial load (copies/mL)
IL-4
NHP-1: R^2 = 0.1088
NHP-3: R^2 = n/a
IL-4 (pg/mL)
Bacterial load (copies/mL)
IL-5
NHP-1: R^2 = 0.9245
NHP-3: R^2 = 0.8533
IL-5 (pg/mL)
Bacterial load (copies/mL)
IL-6
IL-6 (pg/mL)
NHP-1: R^2 = 0.9992
NHP-3: R^2 = 0.0185
Bacterial load (copies/mL)
IL-8
NHP-1: R^2 = 0.0391
NHP-3: R^2 = 0.0446
IL-8 (pg/mL)
Bacterial load (copies/mL)
IL-10
NHP-1: R^2 = n/a
NHP-3: R^2 = n/a
IL-10 (pg/mL)
Bacterial load (copies/mL)
IL-12/23
IL-12/23 (pg/mL)
NHP-1: R^2 = 0.2200
NHP-3: R^2 = 0.4594
Bacterial load (copies/10 mL)
IL-13
NHP-1: R^2 = n/a
NHP-3: R^2 = n/a
IL-13 (pg/mL)
Bacterial load (copies/10 mL)
IL-15
NHP-1: R^2 = 0.0878
NHP-3: R^2 = 0.2841
IL-15 (pg/mL)
Bacterial load (copies/10 mL)
IL-17a
NHP-1: R^2 = n/a
NHP-3: R^2 = n/a
IL-17a (pg/mL)
Bacterial load (copies/10 mL)
IL-18
NHP-1: R^2 = 0.3431
NHP-3: R^2 = 0.0800
IL-18 (pg/mL)
Bacterial load (copies/mL)
MCP-1
MCP-1 (pg/mL)
NHP-1: R^2 = 0.1180
NHP-3: R^2 = 0.2814
Bacterial load (copies/mL)
MIP-1β
NHP-1: R^2 = n/a
NHP-3: R^2 = 0.0377
MIP-1β (pg/mL)
Bacterial load (copies/mL)
MIP-1a
NHP-1: R^2 = n/a
NHP-3: R^2 = n/a
MIP-1a (pg/mL)
Bacterial load (copies/mL)
sCD40L
NHP-1: R^2 = 0.4090
NHP-3: R^2 = 0.0594
sCD40L (pg/mL)
Bacterial load (copies/mL)
TGF-a
TGF-a (pg/mL)
NHP-1: R^2 = 0.5271
NHP-3: R^2 = 0.1061
Bacterial load (copies/mL)
TNF-a
NHP-1: R^2 = n/a
NHP-3: R^2 = n/a
TNF-a (pg/mL)
Bacterial load (copies/mL)
VEGF
NHP-1: R^2 = 0.3732
NHP-3: R^2 = 0.7187
VEGF (pg/mL)
Bacterial load (copies/mL)
Legend
NHP-1
NHP-3

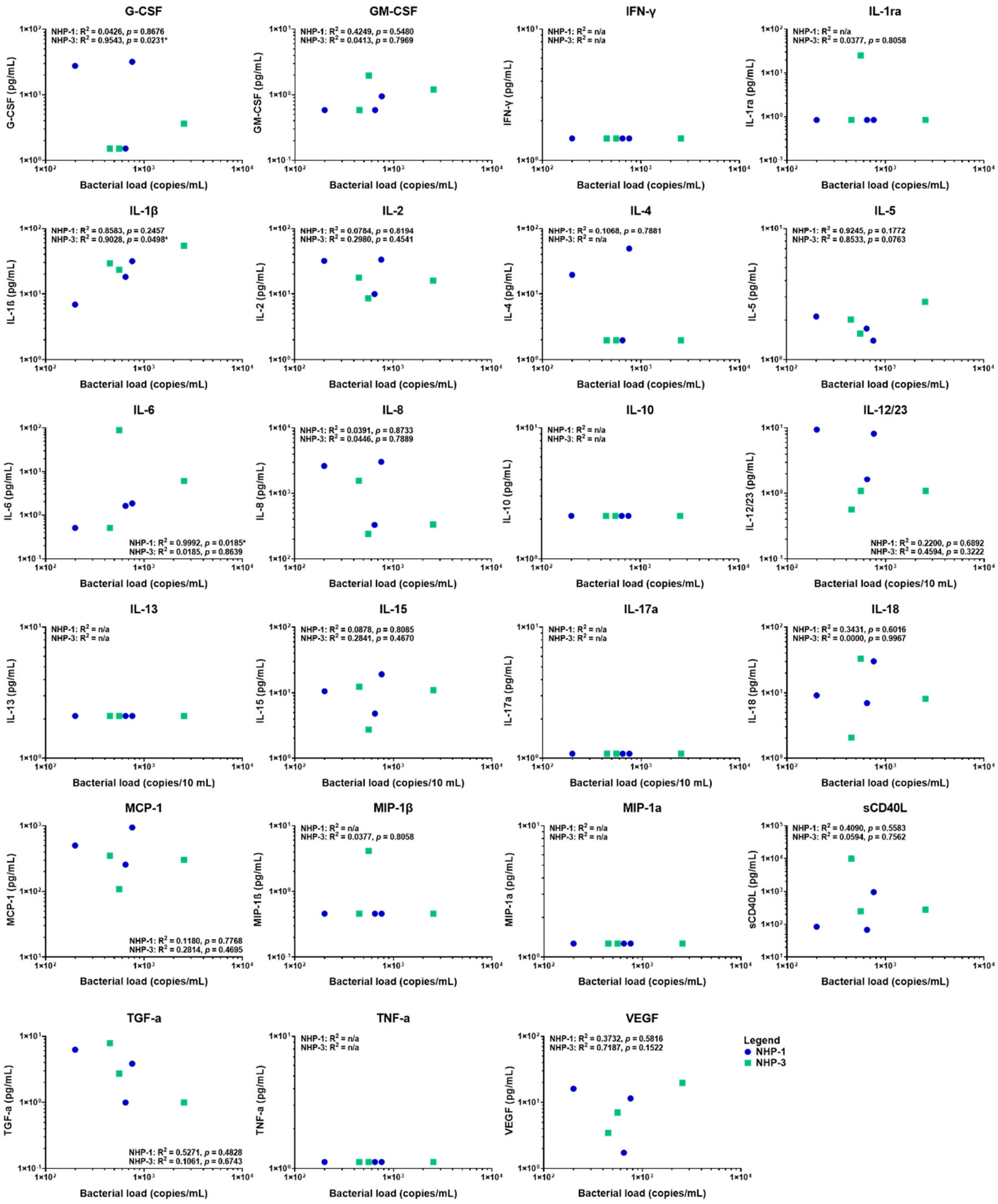

Figure S4. Immunological correlation analysis of serum cytokine/ chemokine of all macaques and bacteremia during acute phase of infection. Correlation efficiency and P value were calculated based on the duplication of each individual samples.
